# Effect of Liners on Pulpal Outcome After Partial Caries Removal in Permanent Teeth: A Systematic Review and Meta-Analysis

**DOI:** 10.7759/cureus.78831

**Published:** 2025-02-10

**Authors:** Vaishnavi R Patankar, Ashish K Jain, Rahul Rao, Prajakta Rao, Deepak Langade, Sanpreet S Sachdev, Krupa Gala

**Affiliations:** 1 Conservative Dentistry and Endodontics, Bharati Vidyapeeth (Deemed to be University) Dental College and Hospital, Navi Mumbai, Mumbai, IND; 2 Periodontology, Bharati Vidyapeeth (Deemed to be University) Dental College and Hospital, Navi Mumbai, Mumbai, IND; 3 Pharmacology, D.Y. Patil University School of Medicine, Navi Mumbai, Mumbai, IND; 4 Oral Pathology and Microbiology, Bharati Vidyapeeth (Deemed to be University) Dental College and Hospital, Navi Mumbai, Mumbai, IND

**Keywords:** cavity liners, deep caries, partial caries removal, pulp vitality, treatment success

## Abstract

Partial caries removal reduces the risk of pulp exposure while preserving the tooth structure that is otherwise unnecessarily lost during the cavity preparation. Liners are applied on the dentinal floors to induce the formation of tertiary dentin that effectively blocks the stimuli from reaching the pulp. The present systematic review was conducted to evaluate the effect of liners on pulp vitality after partial caries removal in permanent teeth. This systematic review followed the resources of evidence-based medicine such as the Cochrane Handbook and was conducted as per PRISMA guidelines. The PICOS strategy was designed and included patients with deep carious lesions in permanent teeth with and without application of liner after partial caries removal, with pulp vitality assessment at one year. After applying the inclusion and exclusion criteria, four articles were selected after an initial list of 750 articles. The results of meta-analysis indicated that there was no statistically significant difference between intervention and control groups (OR=1.440; 95% CL= 0.812 to 2.552; P=0.212). According to the Risk of Bias 2 tool, all four studies were classified as “low risk.” Certainty of evidence was moderate as per GRADE (Grading of Recommendations, Assessment, Development, and Evaluations). Partial caries removal with and without a liner had similar outcomes on pulp vitality at one year follow up. Role of a liner remains controversial in partial caries removal therapy. On the basis of this systematic review and meta-analysis, it may be inferred that treatment success, as defined by continued pulp vitality, is independent of liner application.

## Introduction and background

Many times, there is barely some amount of remaining dentin thickness between the pulp chamber and the active carious lesion. Generally, excavating caries entirely to prepare a conventional cavity can result in pulp exposure, indicating the tooth for root canal treatment [1,2]. Evidence has demonstrated that complete excavation of caries is unwarranted [3]. Preference is, thus, given to the preservation of tooth vitality wherein either the caries is not completely excavated to the extent where it will expose the pulp or a pulp-protecting agent is applied.

A modification of the conventional caries excavation, known as the “Stepwise excavation,” involves leaving a layer of carious dentin over the pulp and temporarily applying a chemical that seals the zone to the external environment. This chemical produces an inductive effect on the pulp stem cells that stimulate dentin formation. Once a sufficient dentin bridge forms between the pulp and the carious front, the carious dentin that was initially left unexcavated is removed after reopening the temporary seal [4,5]. The tooth is then restored with a permanent restorative material. This procedure prevents the pulp from becoming non-vital and also requires root canal treatment [4,5]. It is uncertain if re-entering restorations to remove the residual soft dentin is required, as this might compromise the treatment success [6,7]. Additionally, the technique has certain limitations such as the risk of failure of the temporary seal, and multiple sessions are required, which increase the treatment effort [8,9].

Instead of the complete removal of carious tissue, the current recommendation is "selective or partial removal of carious tissue," which involves the complete removal of carious dentin from cavosurface margins and lateral walls, leaving a layer of soft carious tissue adjacent to pulpal or axial wall [10,11]. This reduces the risk of pulp exposure. Additionally, the procedure can be completed in a single visit as compared to conventional root canal treatment [6,12-15]. This approach has also shown a higher chance of preserving pulp vitality and better caries arrest [13]. According to the AAE Position Statement on Vital Pulp Therapy 2021, histobacteriological studies have consistently revealed the existence of chronic inflammatory cell infiltrates and subclinical pulp inflammation when carious tissues were retained, potentially jeopardizing pulp vitality [16]. Using caries detectors or laser fluorescence during caries removal can help clinicians remove diseased tissues close to the pulp cavity. To enhance pulpal repair, clinicians should focus on removing demineralized infected dentin rather than preventing pulp exposure.

Cavity liners are one such type of pulp-protective coatings that are antibacterial and provide chemical protection [17]. They are used when the remaining dentin thickness is less than 0.5 mm [18]. Various materials are used as pulp protective agents, such as calcium hydroxide, resin-modified glass ionomer, Biodentine™, and mineral trioxide aggregate. Some studies have shown clinical success with the use of different materials as liners after selective caries removal [19-23]. Direct composite restorations (without a liner) placed on carious dentin after selective caries removal have shown positive long-term clinical and radiological outcomes [24-32]. Furthermore, studies have demonstrated that calcium hydroxide liner was not beneficial over direct adhesive restorations after selective caries removal [33,34]. As there are many controversies regarding the role of liners after selective caries removal, the purpose of this systematic review was to analyze the available evidence and provide a detailed analysis of the effect of liners on pulpal outcomes following partial caries removal in permanent teeth.

## Review

Protocol and registration

The present systematic review sought to answer the question “In deep carious lesions, is there any effect of application of a liner on pulpal outcome following partial caries removal in permanent teeth?” The review protocol was conducted according to Preferred Reporting Items for Systematic Review and Meta-analyses (PRISMA) [35]. The protocol was pre-registered in the International Prospective Register of Systematic Reviews (PROSPERO) database with registration no. CRD42022352885.

Eligibility criteria and search strategy

Four databases, PubMed, Cochrane Central Register of Controlled Trials, ScienceDirect, and Google Scholar, were electronically searched by two authors (V.P. and A.J.) using varying combinations of keywords including “selective caries removal,” “partial caries removal,” “partial excavation,” “caries excavation,” and “liner.” Full texts published in the English language since the year 2000 were included. The MeSH terms used in the search are given in Appendix A. The following Population-Intervention-Control-Outcome-Studies (PICOS) criteria were applied“ Population (P) included patients with deep carious lesions in permanent teeth; Intervention (I) was placement of liner after partial caries removal; Comparison (C) was no placement of liner after partial caries removal; Outcome (O) was pulp vitality determined clinically and radiographically after a minimum of one-year follow-up, and study design (S) included only randomized controlled trials (Table 1). Studies published in the English language up to March 2024 were included. Case reports, animal studies, in vitro studies, and review papers were excluded.

**Table 1:** PICOS criteria for inclusion and exclusion of articles in the present systematic review

Parameter	Inclusion criteria	Exclusion criteria
Population (P)	Patients with deep carious lesions in permanent teeth with a diagnosis of reversible pulpitis that required indirect pulp capping	Patients undergoing direct pulp capping or root canal treatment
Intervention (I)	Placement of liner after partial caries removal	Absence of a group in the study for which liner was placed after partial caries removal
Comparison (C)	No placement of liner after partial caries removal (Direct composite restoration)	Absence of a control (no liner) group
Outcome (O)	Pulp vitality determined clinically and radiographically after a minimum of one-year follow-up	Absence of pulp-related outcomes
Study design (S)	Randomized controlled trials	In vitro studies, review articles, animal-based studies, case reports, and case series

Study selection and data extraction

Two reviewers independently examined titles and abstracts to identify relevant studies. Full texts of the potential articles were assessed, and any disagreements were resolved by a third reviewer. A pre-determined Microsoft Excel template was used for extracting data from the selected articles. The extracted data consisted of study characteristics (year, author, design of study, sources of funding), sample characteristics (sample size, age of participants), methodological details (type of liner, test, control, follow up), outcome, and measurement methods (pulp vitality clinically and radiographically).

Risk of bias and certainty of evidence assessment

The quality of the included trials was evaluated by two reviewers independently using the Cochrane Risk of Bias 2 (RoB-2) tool [36]. Every study was categorized according to three risk levels: low, some concerns, and high. The GRADE (Grading of Recommendations, Assessment, Development, and Evaluations) approach was used for assessing the quality of evidence in our systematic review [37].

Data synthesis

The MedCalc Statistical Software Version 19.6.1 was used to conduct the meta-analysis. The findings of each treatment group were pooled together using the odds ratio within the 95% confidence interval (CI) for dichotomous outcomes, such as pulp vitality (success or failure). The effect was judged significant if the p-value was less than 0.05. The Cochran's Q statistic and the I^2^ statistic were used to assess heterogeneity among the included studies [38]. Significant heterogeneity was defined as I^2^ values greater than 50%. A fixed-effects model was employed when no substantial heterogeneity was observed, while the random-effects model was used when considerable heterogeneity was detected. [38] Only studies with similar outcomes were included in the meta-analyses.

Results

Publication Bias

Publication bias could not be assessed by using a funnel plot, as only four studies were included in our meta-analysis. The power of tests is extremely low when there are few studies in order to differentiate chance from actual asymmetry [39].

Study Selection

**Figure 1:**
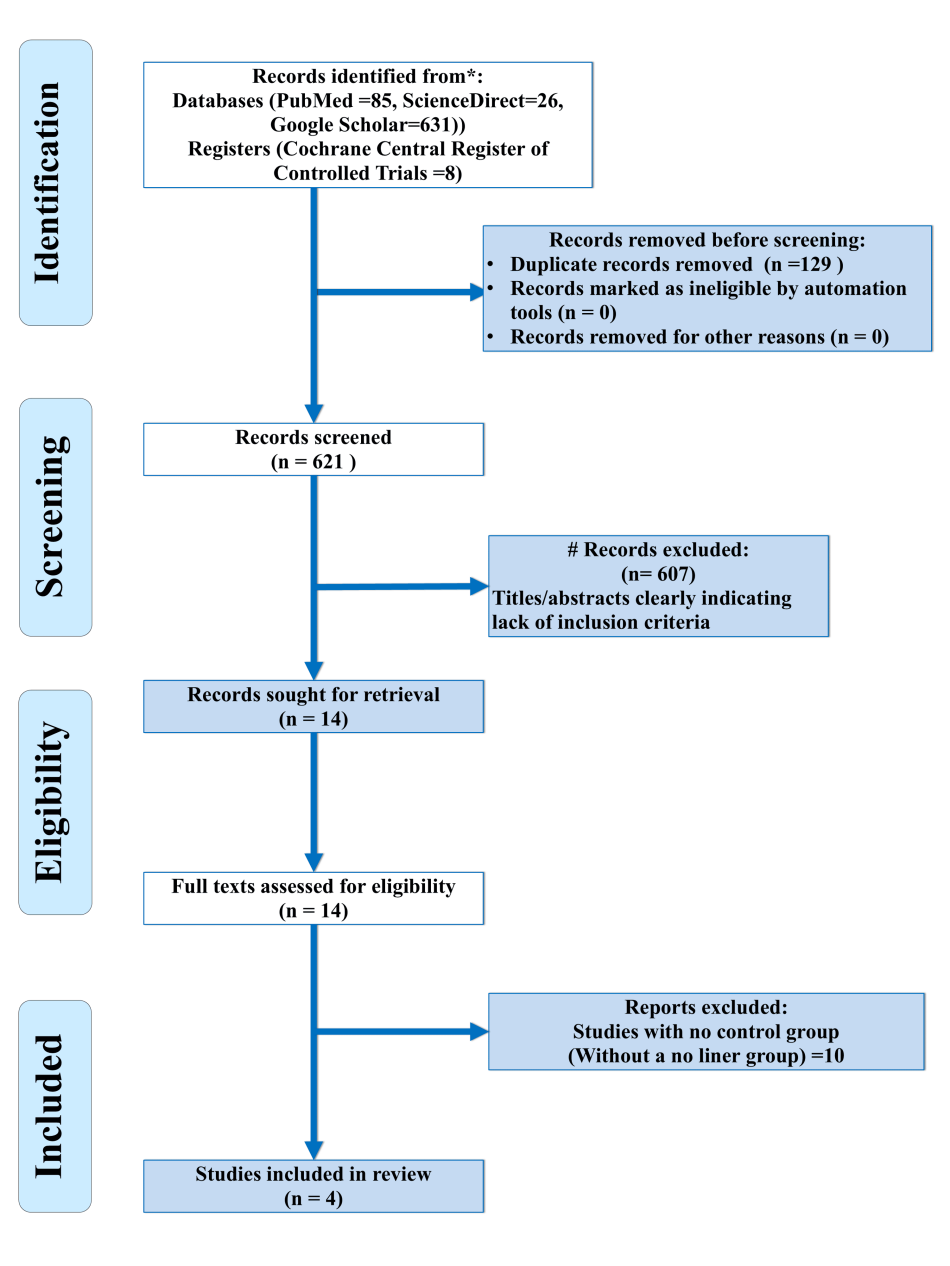
PRISMA flow diagram for study selection in the present systematic review PRISMA, Preferred Reporting Items for Systematic Review and Meta-analyses

A search of the four databases generated 750 results, of which 607 addressed stepwise caries excavation, direct pulp capping, studies on primary teeth, and studies with different outcomes such as microbiologic, ultrastructural, or histologic alterations. Studies with a follow-up of less than a year and studies with unclear methodology were excluded. After the screening of abstracts, 14 full-text articles remained to be examined for eligibility. Of these, 10 articles were excluded after full-text evaluation as they lacked a control (no liner) group. Finally, a total of four articles were included [29-32]. Cohen’s kappa value for the selection of studies was 1, which indicated perfect agreement. Our study selection process is shown in Figure 1.

Study Characteristics

The main characteristics and outcomes of the included studies are summarized in Tables 2, 3. Overall, 334 teeth were included. A total of 167 teeth received liner application and 167 were set as control (no liner). All included studies had primary deep occlusal or occlusal-proximal caries involving at least two-thirds of dentine detected radiographically. Vital teeth with reversibly inflamed pulp and a positive response to cold test without any periapical or periodontal conditions were included. Three studies used light cure glass ionomer cement liner (Torres et al., Singh et al., Banomyong et al.), whereas one study (Gözetici-Çil et al.) used Biodentine as a liner [29-32]. All the studies performed the final restoration of the cavity using composite resin. The included trials assessed pulp vitality after a period of one year. Three out of four trials also assessed the quality of restorations for the same duration of follow-up [29-31]. Pulp vitality was assessed as success or failure by a combination of clinical and radiographic examinations. A positive response to the cold test, a negative response to percussion, and an absence of pain, abscess, or sinus tract were considered clinical success. The lack of periapical disease or alterations such as loss of lamina dura, periodontal ligament space widening, root canal obliteration, and resorption were used to determine radiographic success. In all studies, clinical and radiographic assessments were conducted prior to, immediately following treatment, and at follow-up visits.

**Table 2:** Success and failure with and without liners in the included studies The study by Gözetici-Çil et al. was funded by the Research Fund of Istanbul Medipol University. No other studies received funding. ITT, intention to treat analysis

Study	Success with liner	Success without liner	Failure with liner	Failure without liner	Dropout with liner	Dropout without liner	Follow up (months)	Statistical analysis	Conclusion
Torres et al. [29]	25/25	25/25	0	0	5	5	12	ITT	The application of liner did not influence the clinical performance of composite restorations after partial caries removal in deep carious lesions.
Singh et al. [30]	55/57	53/56	2	3	9	10	12	ITT	Success is independent of the liner used after partial caries removal in deep carious lesions.
										Gözetici-Çil et al. [31]	39/39	36/36	0	0	6	9	12	ITT	The use of liners had no effect on treatment outcome after partial caries removal in deep carious lesions.
Banomyong et al. [32]	23/23	19/19	0	0	3	7	12	ITT	The benefit of placing a liner is questionable after partial caries removal in deep carious lesions.

**Table 3:** Study characteristics of the articles included in the present systematic review RCT, randomized controlled trial

Author and year	Study design	Tooth	Sample size	Age group	Type of liner	Intervention-Liner (number of samples)	Control- No liner (number of samples)	Outcome
Carlos et al. (2020) [29]	RCT (split mouth)	Permanent posteriors	60	38 ± 5.64 years	Light cured Glass ionomer composite liner (Ionoseal, Voco)	30	30	Pulp vitality
									Singh et al. (2019) [30]	RCT (parallel)	Permanent molars	132	14-54 years	RMGIC (GC II Fuji Lining LC)	66	66	Pulp vitality
Gözetici-Çil et al. (2022) [31]	RCT	Permanent posteriors	90	13-44 years	Biodentine™	45	45	Pulp vitality
Banomyong et al. (2011) [32]	RCT	Permanent molars	52	18–40 years	Glass ionomer cement (Fuji Lining LC)	26	26	Pulp vitality

Risk of Bias

**Figure 2:**
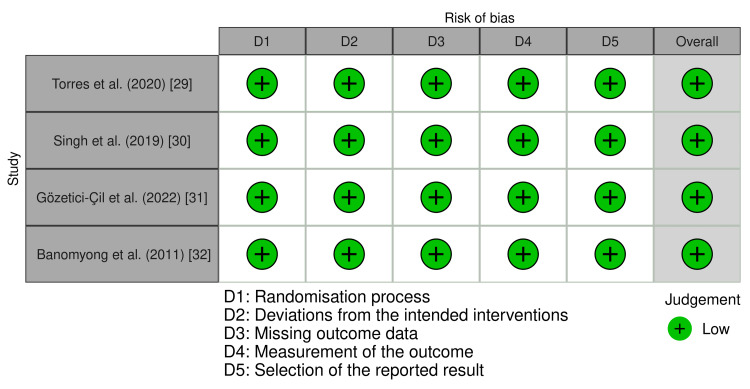
Risk-of-bias assessment according to the RoB 2 tool

The risk-of-bias assessment is presented in Figure 2. All four trials gave a clear idea regarding the process of randomization. Allocation concealment was clearly addressed in all trials. Regarding the blinding of participants and the operator, it was impossible to conceal the treatment method from the operator. Blinding to outcome assessment was clear in all trials. No trials had deviations arising from missing data. Overall, all four studies were classified as low-risk [29-32].

Certainty of Evidence Assessment

The quality of evidence was graded as Moderate. The true effect was likely to be close to the estimate of the effect. Only randomized trials, which provide high-quality data, were used as the study design. Due to effective blinding, allocation concealment, and comprehensive reporting of results, the risk of bias was not serious. Heterogeneity across studies was 0.00%, and, thus, inconsistency was not serious. The results of studies have direct evidence (no indirectness). Population, intervention, and outcome measures did not significantly vary between trials. Because the median sample size was small and there were few included studies, the degree of imprecision was rated as serious. Publication bias could not be determined as there were only four studies. The evaluation of the quality of evidence is summarized in Table 4.

**Table 4:** Evaluation of certainty of evidence of the articles included in the present review using the GRADE approach GRADE, Grading of Recommendations, Assessment, Development, and Evaluations

Author (year)	Study design	Risk of Bias	Inconsistency	Indirectness	Imprecision	Publication bias	Certainty
Torres et al. [29]	Randomized controlled trial	Not serious	Not serious	Not serious	Serious	Undetected	Moderate ⊕⊕⊕◯
Singh et al. [30]	Randomized controlled trial	Not serious	Not serious	Not serious	Serious	Undetected	Moderate ⊕⊕⊕◯
Gözetici-Çil et al. [31]	Randomized controlled trial	Not serious	Not serious	Not serious	Serious	Undetected	Moderate ⊕⊕⊕◯
Banomyong et al. [32]	Randomized controlled trial	Not serious	Not serious	Not serious	Serious	Undetected	Moderate ⊕⊕⊕◯

Meta-Analysis

Out of 167 teeth in both groups, 142 had success with liner and 132 had success without liner. Meta-analysis was performed with four studies, and the odds ratio was calculated. The odds ratio of 1.4 was obtained by analyzing success data in both groups (Table 5). The odds of success with the application of liner were 14% more than that of success without liner. However, according to both the random and fixed effect models, a p-value of greater than 0.05 was obtained, which indicated no significant difference between the intervention and control groups. Since the heterogeneity was calculated to be I^2 ^= 0% (p=0.74), the fixed effects model was preferred.

**Table 5:** Meta-analysis of the studies assessing success rates with and without the application of liners

Study	Intervention	Controls	Odds ratio	95% CI	z	p	Weight (%)	
							Fixed	Random
Torres et al. [29]	25/30	25/30	1.000	0.257 to 3.888	-	-	17.76	17.76
Singh et al. [30]	55/66	53/66	1.226	0.505 to 2.978	-	-	41.61	41.61
Gozetici-Cil et al. [31]	39/45	36/45	1.625	0.526 to 5.020	-	-	25.74	25.74
Banomyong et al. [32]	23/26	19/26	2.825	0.641 to 12.442	-	-	14.90	14.90
Total (fixed effects)	142/167	133/167	1.450	0.823 to 2.556	1.285	0.199	100.00	100.00
Total (random effects)	142/167	133/167	1.440	0.812 to 2.552	1.249	0.212	100.00	100.00

**Figure 3:**
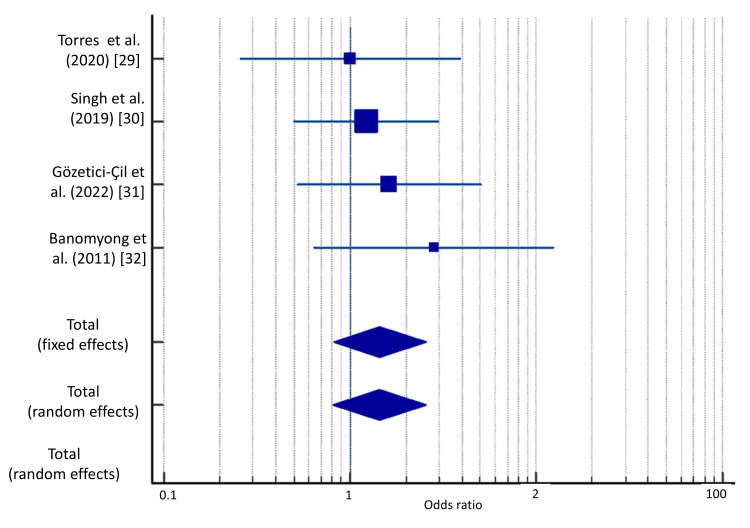
Forest plots and results of the meta-analysis

The results of the meta-analysis are shown in Figure 3. The horizontal line represents each study. The width of the line represents the range of the confidence interval. The box on each study’s line represents the point estimate of that study. The size of the box represents the “weight” of the study. The larger the study, the larger the box. The diamond represents the pooled effect estimates and confidence intervals from all studies included in the meta-analysis. The vertical line represents the line of null effect. As the diamond crosses the line of null effect, there is no statistical difference between the application of a liner and direct composite restoration without a liner among all trials in the meta-analysis with an odds ratio of 1.4.

Discussion

The present systematic review and meta-analysis aimed to determine the clinical outcomes following liner application after partial caries removal. The findings supported the notion that there was no significant difference in the outcomes related to pulp vitality whether or not a liner was applied following partial excavation of caries. Similar results were observed across studies included in a systematic review conducted on pulp vitality outcomes after liner application in primary teeth [40-42]. Partially removing the carious dentin on the pulpal wall and obtaining a good interfacial seal by a proper restoration are recommended measures to halt the progression of the lesion and decrease dentin permeability via sclerosis and tertiary dentin formation [43]. When the source of nutrition (carbohydrates in the oral cavity) that the organisms within carious lesions need to survive is eliminated by a good coronal seal, both the quantity and diversity of microorganisms diminish, thereby initiating a healing and self-repair process [43]. Evidence of biochemical and radiographic remineralization of residual carious dentine over a time period of seven months has been provided by researchers [44].

One study used wax as a control to examine the effects of calcium hydroxide and glass ionomer cement on carious dentin following partial caries removal and sealing. They observed dentin hardness, better dentin organization, full or partial obliteration of dentinal tubules, and a decrease in bacterial load in all groups with no statistically significant differences, regardless of the liner utilized [28]. Another study examined the efficacy of universal adhesive, followed by composite restoration, and calcium hydroxide liners in preserving pulp vitality following selective caries removal. They came to the conclusion that lining material had no bearing on treatment success as there were no statistically significant differences between the two groups [27].

According to a study, the number of microbes found following partial caries removal and sealing for three months and complete caries removal did not differ [45,46]. Nutritional starvation is the most evident environmental challenge for the remaining microbes [46]. On the other hand, sealed carious dentine was found to be less infected than the dentine that remained after traditional caries removal and sealing. Microbial persistence does not appear to be a factor for reopening cavities in permanent teeth following partial caries treatment [45]. The pulp responds constructively in such events rather than undergoing degeneration forming reparative and sclerotic dentin. The only exceptions are in cases of genuine penetration of the pulp chamber by instruments or microbes wherein the possibility of occurrence of degenerative processes increases [47]. Selective or partial caries removal, independent of liner application, successfully treats deep carious lesions in permanent teeth. Impediment in bacterial metabolism by eradicating the necrotic dentine and isolating bacteria from the oral milieu by a decent coronal seal decreases caries advancement, thus allowing the defensive responses of the dentine-pulp complex [45].

The studies included in our systematic review evaluated pulp vitality-based clinical criteria, such as the presence of symptoms, and response to cold tests. Additional confirmation by radiographs was also performed in all the studies. Cold tests are not completely reliable, and a definitive diagnosis can only be achieved after a histologic examination. Thus, the information collected should be interpreted carefully as teeth with pulp necrosis could be asymptomatic. Also, teeth that are responsive may have portions of pulp that are irreversibly damaged [47]. Hence, it is likely that an incorrect diagnosis would have led to bias. It would be better to use a combination of tests for diagnosis and evaluation of outcome such as an electric pulp test along with a cold test until methods with greater specificity and sensitivity are adopted. Another suggestion that can be considered while designing future trials is to define the volume of residual caries left. None of the studies quantified the residual volume or thickness of caries. The amount of soft caries remaining over the pulp could affect the pulp vitality and restoration survival. Additional variables such as the hardness of dentin (soft, firm, or leathery) along with removal methods (micromotor, spoon excavator, turbine) should be standardized [3]. According to a systematic review of studies in primary teeth, selective caries removal to soft dentin may increase restoration failure [41]. The demineralized dentin could reduce adequate bonding to overlying restoration. Thus, it can be suggested to recall patients at shorter intervals to evaluate restoration quality and control caries.

The strengths of this systematic review include a thorough literature search conducted, high-quality evidence randomized trials, all studies with a low risk of bias, no statistical heterogeneity across studies, and all studies with moderate certainty of evidence. The small number of studies included in qualitative and quantitative analysis is one of the potential limitations of this review. Because all studies included in this analysis employed different liner materials, it would be prudent to include data from trials that used one particular liner. Another drawback is that the review only included papers in English, which may have resulted in insufficient conclusions. Only those studies that measured success using radiographs were included. Future studies must take into account additional diagnostic methods such as CBCT. Due to the low number of studies, no statistical significance was observed in the meta-analysis, even with 14% more success with liners. However, due to an apparent paucity of research, meta-analysis for other crucial clinical parameters such as restoration survival and postoperative sensitivity with and without liners could not be conducted. The level of research and number of studies included in this systematic review suggests that more randomized clinical trials with larger sample sizes, standardized outcome measurement methods, and longer follow‐up periods must be conducted to evaluate the effect of liners on pulp vitality, restoration quality, and longevity following partial caries removal in permanent teeth.

## Conclusions

Within the limitations of this systematic review and meta-analysis, it can be concluded that the application of liners following partial caries removal in permanent teeth does not significantly influence pulp vitality outcomes at one-year follow-up. The findings suggest that pulp preservation and treatment success are primarily dependent on achieving a proper coronal seal rather than the use of a liner. While liners may provide additional benefits such as chemical protection and antibacterial properties, their clinical necessity in partial caries removal remains uncertain. The current evidence, graded as moderate, highlights the need for further well-designed randomized controlled trials with larger sample sizes, standardized methodologies, and longer follow-up periods to establish definitive clinical guidelines regarding liner application. Additionally, future research should focus on evaluating other critical parameters, such as restoration longevity, patient-reported outcomes, and cost-effectiveness, to better inform clinical decision-making.

## Appendices

Appendix A

**Table 6:** MeSH terms used for performing search in the present systematic review across various databases

Database	Search strategy	Findings
Medline (PubMed) Filters applied: Clinical Trial, Randomized Controlled Trial.	(“Partial Caries Removal”) AND (“Dental Caries”) AND (“Dental Cavity Liner” OR “Liners”) AND (“Dental Pulp” OR “Pulp”) AND (“Vitality”) AND (“Selective Caries Removal”) AND (“Partial Caries Removal”) AND (“Treatment Success” OR “Success”) AND (“Treatment Outcome”) AND (“Deep Caries”) AND (“Deep Caries Management”)	7
	(“Partial Caries Removal” OR “Excavation”) AND (“Dental Cavity Liner” OR “Liners”) AND (“Pulp”) AND (“Vitality”)	5
	(“Selective Caries Removal”) AND (“Treatment Outcome”) AND (“Deep Caries”)	12
	(“Treatment Outcome”) AND (“Deep Caries”)	61
Science direct Filters applied: Research articles	“Cavity Liners”, “Pulp Vitality”, “Partial Caries Removal”, “Deep Caries”, “Treatment Success”, “Outcome”	26
Google scholar	“Dental Cavity Liners”, “Pulp Vitality”, “Partial Caries Removal” OR “Excavation”, “Deep Caries”, “Treatment Success” OR “Outcome”, “Randomized Controlled Trial”	631
Cochrane Central Register of controlled trials	“Partial Caries Removal”, “Cavity Liners”	8
